# Metagenomic Analysis Identifies Sex-Related Cecal Microbial Gene Functions and Bacterial Taxa in the Quail

**DOI:** 10.3389/fvets.2021.693755

**Published:** 2021-10-01

**Authors:** Jing-E Ma, Xin-Wei Xiong, Ji-Guo Xu, Ji-Shang Gong, Jin Li, Qiao Xu, Yuan-Fei Li, Yang-Bei Yang, Min Zhou, Xue-Nong Zhu, Yu-Wen Tan, Wen-Tao Sheng, Zhang-Feng Wang, Xu-Tang Tu, Cheng-Yao Zeng, Xi-Quan Zhang, You-Sheng Rao

**Affiliations:** ^1^Institution of Biological Technology, Nanchang Normal University, Nanchang, China; ^2^Jiang Xi Province Key Lab of Genetic Improvement of Indigenous Chicken Breeds, Nanchang, China; ^3^Guangdong Provincial Key Lab of Agro-Animal Genomics and Molecular Breeding, Guangzhou, China; ^4^Key Lab of Chicken Genetics, Breeding and Reproduction, Ministry of Agriculture, Guangzhou, China

**Keywords:** quail, gut microbiome, metagenomic analysis, reference genes, cecal bacteria

## Abstract

**Background:** Japanese quail (*Coturnix japonica*) are important and widely distributed poultry in China. Researchers continue to pursue genetic selection for heavier quail. The intestinal microbiota plays a substantial role in growth promotion; however, the mechanisms involved in growth promotion remain unclear.

**Results:** We generated 107.3 Gb of cecal microbiome data from ten Japanese quail, providing a series of quail gut microbial gene catalogs (1.25 million genes). We identified a total of 606 main microbial species from 1,033,311 annotated genes distributed among the ten quail. Seventeen microbial species from the genera *Anaerobiospirillum, Alistipes, Barnesiella*, and *Butyricimonas* differed significantly in their abundances between the female and male gut microbiotas. Most of the functional gut microbial genes were involved in metabolism, primarily in carbohydrate transport and metabolism, as well as some active carbohydrate-degrading enzymes. We also identified 308 antibiotic-resistance genes (ARGs) from the phyla Bacteroidetes, Firmicutes and Euryarchaeota. Studies of the differential gene functions between sexes indicated that abundances of the gut microbes that produce carbohydrate-active enzymes varied between female and male quail. Bacteroidetes was the predominant ARG-containing phylum in female quail; Euryarchaeota was the predominant ARG-containing phylum in male quail.

**Conclusion:** This article provides the first description of the gene catalog of the cecal bacteria in Japanese quail as well as insights into the bacterial taxa and predictive metagenomic functions between male and female quail to provide a better understanding of the microbial genes in the quail ceca.

## Introduction

Japanese quail (*Coturnix japonica*), from the order Galliformes and family Phasianidae, are an important poultry species for egg and meat production and are widely distributed in China. Quail have a short maturation period, and female quail can lay their first eggs at 35 days old. The laying rate can reach 50% at 45 days, and the weight of annual egg production is an average of 20–25 times that of the female quail's body weight. Quail make ideal animal models owing to their small body size and short generation intervals. Quail byproducts also have commercial applications, for example, feathers in duvet manufacturing. Therefore, some researchers consider quail breeding to be the future of twenty-first century poultry breeding (1, 2).

The gut microbiota plays an important role in production and disease resistance in many animals, especially in digestion and nutrient absorption, contributing to feed-related traits (3–10). 16S rRNA genes were analyzed to profile the cecal bacterial communities of chicken; these analyses showed that the male animals had high abundances of *Bacteroides*, whereas female animals were enriched with *Clostridium* and *Shigella*. Microbiome-wide association analyses in the ilea and ceca of Japanese quail have shown that several quantitative feed-related traits, including feed or nutrient efficiency, feed intake, body weight gain, feed per gain ratio, and phosphorus utilization were associated with microbiota features at both the bacterial genus and operational taxonomic unit levels as characterized by 16S rRNA amplicon sequencing (11–16). Beneficial groups of bacteria (i.e., probiotics) in the gut help balance the intestinal flora and improve the body's disease resistance via competitive inhibition of acid substance production. *Bacillus* and some subspecies of *Enterococcus* are considered probiotics in chickens and Japanese quail (17, 18). However, to our knowledge, the reference gene catalogs of the gut microbiome, including the intestinal bacterial compositions and the functional capacity of the gut microbiome, have rarely been applied or reported for Japanese quail. This could hinder growth promotion in quail.

Female quail exhibit greater growth potential in the breast, wings and back than do male quail for both yellow and red laying Japanese quail, but no relevant research is available on the gut microbiotas of adult quail in China (1, 2). Variations in growth rates between the sexes might be due to differences in their gut microbiomes because the gut microbiome has key effects on nutrient digestion, absorption, and metabolism in quail (19, 20). However, knowledge of how the gut microbiome varies between sexes in quail is minimal. This study was conducted to examine how nutrient digestion and absorption by the gut microbiome differ between male and female quail and whether any gut bacteria are beneficial to quail.

Here, we used shotgun metagenomic sequencing to analyze the microbiomes from cecal samples of ten 70-day-old Japanese quail. At this age, the cecal microbiota should be mature and stable (21). We compared the gut microbiotas between female and male quail to study the gut microbial ecosystem and the differences in the gut microbiotas between the sexes in this economically important species.

## Materials and Methods

### Sample Collection

Samples were collected from the cecal contents of ten 70-day-old Japanese quail (one sample per quail), immediately frozen in liquid nitrogen, and stored at −80°C until DNA extraction. We used five healthy adult female quail and five healthy adult male quail. All birds were offered the same diet and housed under similar environmental conditions. Supplementary Table 1 provides the details of the samples.

DNA was extracted using the Magen DNA Stool Kit per the manufacturer's protocol (Magen, Guangdong, China), using 200 mg of feces per sample. The DNA samples were tested using two methods. First, the degree of DNA degradation was determined on 1% agarose gels, and second, the DNA concentration was measured using a Qubit^®^ dsDNA Assay Kit in a Qubit^®^ 2.0 Fluorometer (Life Technologies, CA, USA). The optical density was between 1.8 and 2.0. The DNA contents were used to construct a sequencing library.

### Library Construction

One microgram of DNA per sample was used as the input material to prepare the DNA samples. Sequencing libraries were generated using NEBNext^®^ Ultra^™^ DNA Library Prep Kit for Illumina (NEB, USA), and recommendations and index codes were added to attribute sequences to each sample. Briefly, the DNA sample was fragmented by sonication to 350 bp, then the DNA fragments were end-paired, poly-A-tailed, and ligated with the full-length adaptor for Illumina sequencing with further PCR amplification. PCR products were then purified (AMPure XP system), and libraries were analyzed for size distribution using an Agilent 2100 Bioanalyzer and quantified using real-time PCR.

### DNA Sequencing and DNA Assembly

Index-coded samples were clustered using a cBot Cluster Generation System per the manufacturer's instructions. After cluster generation, the library preparations were sequenced on an Illumina HiSeq platform, and paired-end reads were generated.

After preprocessing the raw data, clean data were obtained using subsequent analyses, which included removing reads that contained low-quality bases (default quality threshold value ≤ 38) above a certain portion (default length: 40 bp), removing reads in which the N base had reached a certain percentage (default length: 10 bp), and removing reads that overlapped by more than a certain portion using Adapter (default length: 15 bp). To remove the effects of host pollution, clean data were analyzed using Basic Local Alignment Search Tool (BLAST) against the Japanese quail genome database (INSDC: LSZS01000000), which defaults to using Bowtie2.2.4 software (Bowtie2.2.4, http://bowtiebio.sourceforge.net/bowtie2/index.shtml) to filter reads of host origin using the following parameters (22, 23): –end-to-end, –sensitive, -I 200 and -X 400.

### Construction of the Gene Catalog and Abundance Analysis

To construct a comprehensive Japanese quail gut microbial gene catalog, all reads were assembled *de novo* from each sample into longer contigs, which were assembled and analyzed (24) using SOAPdenovo software (V2.04, http://soap.genomics.org.cn/soapdenovo.html), with the parameters -d 1, -M 3, -R, -u, -F and -K 55 (25–28). The assembled scaffolds were then broken from the N junction to acquire scaffolds not containing N (called scaftigs) (26, 29, 30). The clean data were compared with each respective scaffold using Bowtie2.2.4 to acquire unused paired-end reads with the parameters –end-to-end, –sensitive, -I 200 and -X 400 (26). Fragments shorter than 500 bp were filtered from all scaftigs because of the lengths of the assembly sequences.

The assembled scaftigs (≥500 bp) were used to predict the open reading frames (ORFs) using MetaGeneMark (V2.10, http://topaz.gatech.edu/GeneMark/) software, then the sequences with coding region lengths <100 nt were filtered from the predicted results using the default parameters (26, 30–33). CD-HIT software (34, 35) (V4.5.8, http://www.bioinformatics.org/cd-hit) was used to obtain the unique initial gene catalog (the genes referred to here are the nucleotide sequences coded by unique and continuous genes) (36), with the parameters -c 0.95, -G 0, -aS 0.9, -g 1, and -d 0 (33, 36). Each gene's abundance was calculated from the number of mapped reads and the gene length. The formula used was $G_{k}=\frac{r_{k}}{L_{k}}\cdot \frac{1}{\sum_{i=1}^{n}\frac{r_{i}}{L_{i}}}$, where r represents the number of reads mapped to the genes, and L represents gene length (31).

The gene numbers were analyzed from the abundance of each gene in each sample in the gene catalog; the analyses included the basic statistical information, core-pan gene analysis, correlation analysis of the samples and a Venn diagram.

All predicted unigenes were analyzed in BLAST against functional databases with the parameters blastp and -e 1e-5 using DIAMOND software (V0.9.9; https://github.com/bbuchfink/diamond/), with the exceptions of the Kyoto Encyclopedia of Genes and Genomes [KEGG; (37, 38); version 2018-01-01, http://www.kegg.jp/kegg/], EggNOG (39); version 4.5, http://eggnogdb.embl.de/#/app/home), and Carbohydrate-Active Enzymes [CAZy; (40) version 201801, http://www.cazy.org/] databases. For each sequence's BLAST result, the best BLAST hit was used for subsequent analyses (28, 32, 41). To annotate the resistance genes, Resistance Gene Identifier software was used to align the unigenes to the CARD database (https://card.mcmaster.ca/) (42–44), using the parameters blastp and e-value ≤ 1e^−30^. The relative abundances of antibiotic-resistance ontology genes from the aligned results were counted.

### Taxonomic Assignment of Genes and Construction of Taxonomy and Relative Abundance Profiles

DIAMOND [28] software (V0.9.9) was used to analyze the unigenes via BLAST against the bacterial, fungal, archaeal and viral sequences, which were extracted from the NR database (Version: 2018-01-02, https://www.ncbi.nlm.nih.gov/) of the National Center for Biotechnology Information with the parameter settings blastp and -e 1e^−5^. Because each sequence could have multiple aligned results, sequences were chosen for alignment if the E-value was ≤ the smallest E-value × 10 (45). The least common ancestors (LCA) algorithm was also applied to the system classification using MEGAN software (46) to confirm the species annotation.

The number of genes and the abundance information for each sample were obtained using the LCA annotation results and the gene abundance for each taxonomic hierarchy (kingdom, phylum, class, order, family, genus, and species). The species abundance in one sample equaled the sum of the gene abundances annotated for the species. The gene number of a species in a sample equaled the number of genes with abundances >0.

These analyses were based on the abundance results for each taxonomic hierarchy, including the relative abundance and abundance cluster heat maps. Differences between two groups were tested via analysis of similarity (ANOSIM; R vegan package, version 2.15.3). Metastats and linear discriminant analysis effect size (LEfSe) analysis were used to detect different species between groups. Permutation tests between groups were conducted in Metastats for each taxonomic level to obtain the *p*-value, then the Benjamini and Hochberg false discovery rate procedure was used to correct the *p*-value and acquire a *q*-value (47). LEfSe analysis was conducted using LEfSe software, with a default LDA score of 3. Important species were screened out via MeanDecreaseAccuracy and MeanDecreaseGini, and the receiver operating characteristic curve was plotted to cross validate each model (default: 10 times).

## Results

### Microbial Genome Sequencing and *de novo* Metagenome Assembly

We sequenced 107.3 Gb of high-quality data, with an average of 10.73 Gb per sample (SRA accession: SUB9360165) after removing low-quality reads, N reads and adaptor sequences. This included 99.5 Gb of no-host data. Table 1 lists the raw, clean and preprocessed data. The best assembly with different k-mer sizes was chosen on the basis of contig N50 and the mapping rate. The lengths of the contig N50 in each sample ranged from 1,662 to 4,217 bp. Table 2 summarizes the assembly results. The length distribution of the contigs ranged from 500 to 3,000 bp, with most of the length distribution falling between 500 and 1,000 bp (Supplementary Figure 1).

**Table 1:** The statistics of the raw data, clean data and preprocessed data.

	**InsertSize (bp)**	**SeqStrategy**	**RawData**	**RawReads (#)**	**N_num**	**CleanData**	**Clean_Q20**	**Clean_Q30**	**Clean_GC (%)**	**Effective %)**	**Non-HostData**
F1	350	(150:150)	11,003.63	73,357,520	0.01	11,003.60	97.13	92.29	48.7	100	9,074.26
F2	350	(150:150)	10,712.92	71,419,466	0.01	10,712.91	97.44	93	46.82	100	8,856
F3	350	(150:150)	10,687.25	71,248,310	0.21	10,686.86	97.44	92.7	47.96	99.996	10,675.16
F4	350	(150:150)	10,323.51	68,823,430	0.1	10,323.33	97.36	92.73	48.25	99.998	9,213.76
F5	350	(150:150)	10,744.70	71,631,314	0.02	10,744.66	97.58	93.06	47.91	100	10,741.30
M1	350	(150:150)	10,817.98	72,119,874	0.23	10,817.55	97.58	93.13	52.16	99.996	10,803.80
M2	350	(150:150)	11,251.57	75,010,482	0.23	11,251.14	97.7	93.36	49.58	99.996	11,249.04
M3	350	(150:150)	10,449.38	69,662,506	0.02	10,449.34	97.42	92.54	46.84	100	9,998.72
M4	350	(150:150)	10,283.92	68,559,476	0.08	10,283.77	97.54	93.04	52.79	99.998	10,278.34
M5	350	(150:150)	11,037.03	73,580,196	0.08	11,036.87	97.74	93.5	52.55	99.999	10,953.34

**Table 2:** A summary of the assembly results.

**SampleID**	**Total len.(bp)**	**Num**.	**Average len.(bp)**	**N50 Len.(bp)**	**N90 Len.(bp)**	**Max len.(bp)**
M1	320,557,884	206,569	1,551.82	2,124	635	316,802
M2	284,615,154	174,941	1,626.92	2,326	648	330,539
M3	203,706,984	132,003	1,543.20	2,064	636	285,679
M4	284,104,470	198,983	1,427.78	1,767	615	468,579
M5	304,165,913	207,528	1,465.66	1,890	623	294,780
F1	300,816,819	217,776	1,381.31	1,662	621	511,013
F2	216,638,880	133,104	1,627.59	2,384	641	458,696
F3	276,148,607	134,475	2,053.53	4,217	706	357,891
F4	300,219,893	188,990	1,588.55	2,269	637	464,221
F5	300,697,034	195,495	1,538.13	2,089	629	317,717

### Gut Microbiome Gene Catalog Prediction and Taxonomic Annotation

Based on the scaftigs, 3,378,594 ORFs were constructed, and core-pan gene analysis showed that the curve approached saturation (Supplementary Figure 2). We obtained 1,247,092 ORFs after reducing the data redundancy; 48.76% of these were complete ORFs, and 608,045 genes were obtained. The female quail contained 303,162 common ORFs; the male quail contained 401,051 common ORFs (Supplementary Figure 3).

Using BLAST against the MicroNR database, we annotated 1,033,311 genes from 1,247,092 non-redundant genes. Of these, 0.59% were annotated as viruses, 0.0364% as eukaryotes, 0.203% as archaea, and 13.9% as unknown. There were 382, 142, 175, and 7,047 species belonged to the kingdom of the viruses, eukaryotes, archaea, and bacteria, respectively (Supplemental File 1). The remaining 88.74% of the genes were annotated as bacteria at the kingdom level, with 85.27% annotated at the phylum level, 81.27% at the class level, 80.45% at the order level, 68.07% at the family level, 63.42% at the genus level, and 45.33% at the species level. Of the 1,033,311 annotated genes in MicroNR, many strains belonged to the phyla Bacteroidetes, Firmicutes, Proteobacteria, Spirochaetes, and Fusobacteria.

The most abundant genera were *Bacteroides* (mean abundance 24.9), *Alistipes* (mean abundance 4.48), *Prevotella* (mean abundance 3.07), *Mediterranea* (mean abundance 3.14), *Lachnoclostridium* (mean abundance 2.67), *Flavonifractor* (mean abundance 2.62), and *Barnesiella* (mean abundance 1.10; Supplemental File 2). A series of genes were also annotated to *Bacillus* and *Enterococcus* (Supplemental File 3).

From the ten quail tested, we identified 4 Kingdom, 129 Phylum, 115 Class, 240 Order, 510 Family, 1,857 Genus, and 7,746 microbial species. We detected 606 bacterial species with a higher abundances from the ten quail (Supplemental File 4): 7,302 from the male quail, with each individual containing 6,073, 5,910, 5,468, 5,932, and 6,089 species, and 7,573 from the female quail, with each individual containing 6,245, 5,234, 5,075, 6,463, and 6,017 species.

**Figure 1:**
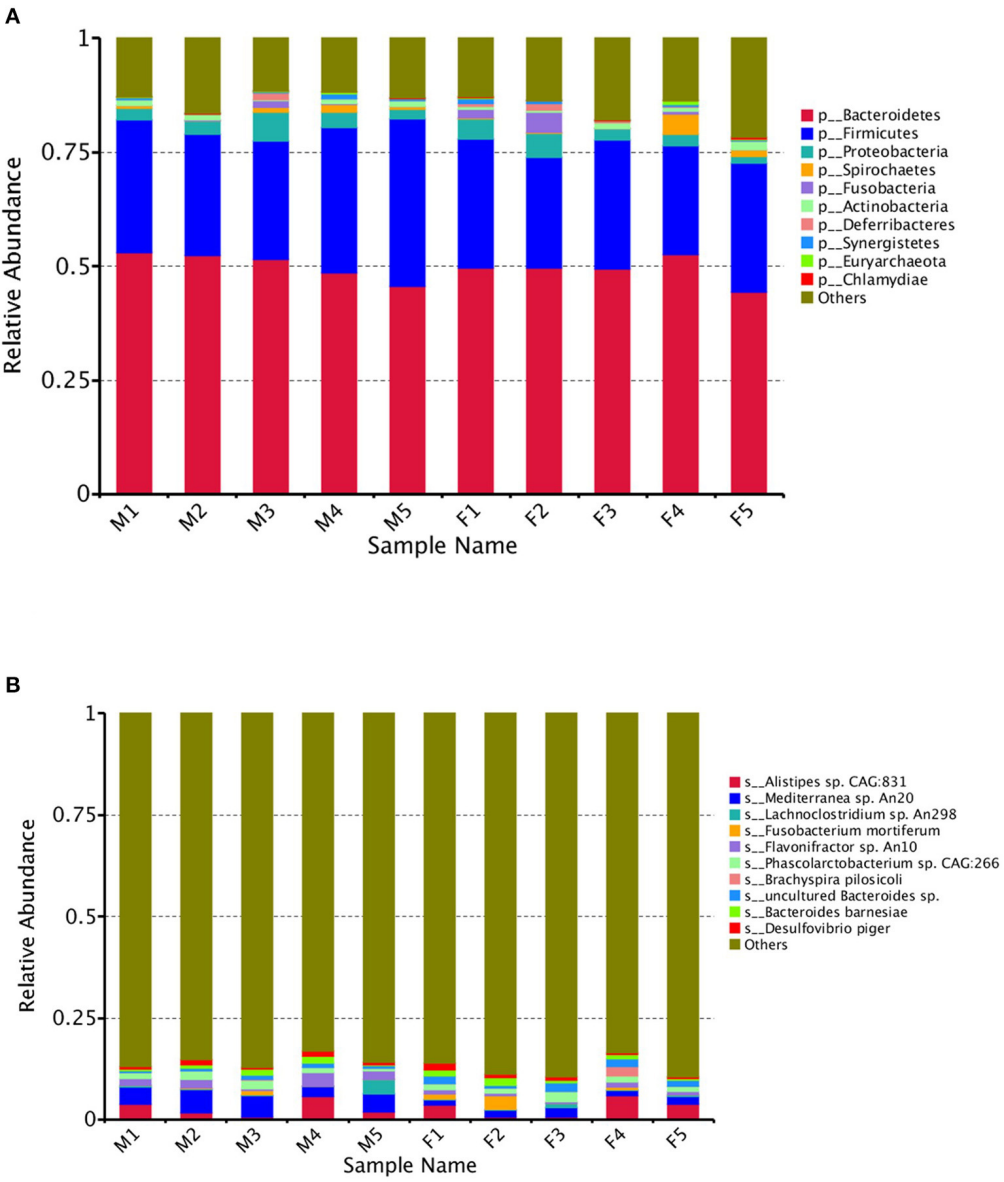
The dominant microbial species in female (F) and male (M) Japanese quail (*Coturnix japonica*) at the phylum **(A)** and species **(B)** levels.

The gene abundances revealed several dominant microbial species in both groups at different levels. The top five were assigned to Bacteroidetes, Firmicutes, Proteobacteria, Spirochaetes, and Fusobacteria (Figure 1A).

**Figure 2:**
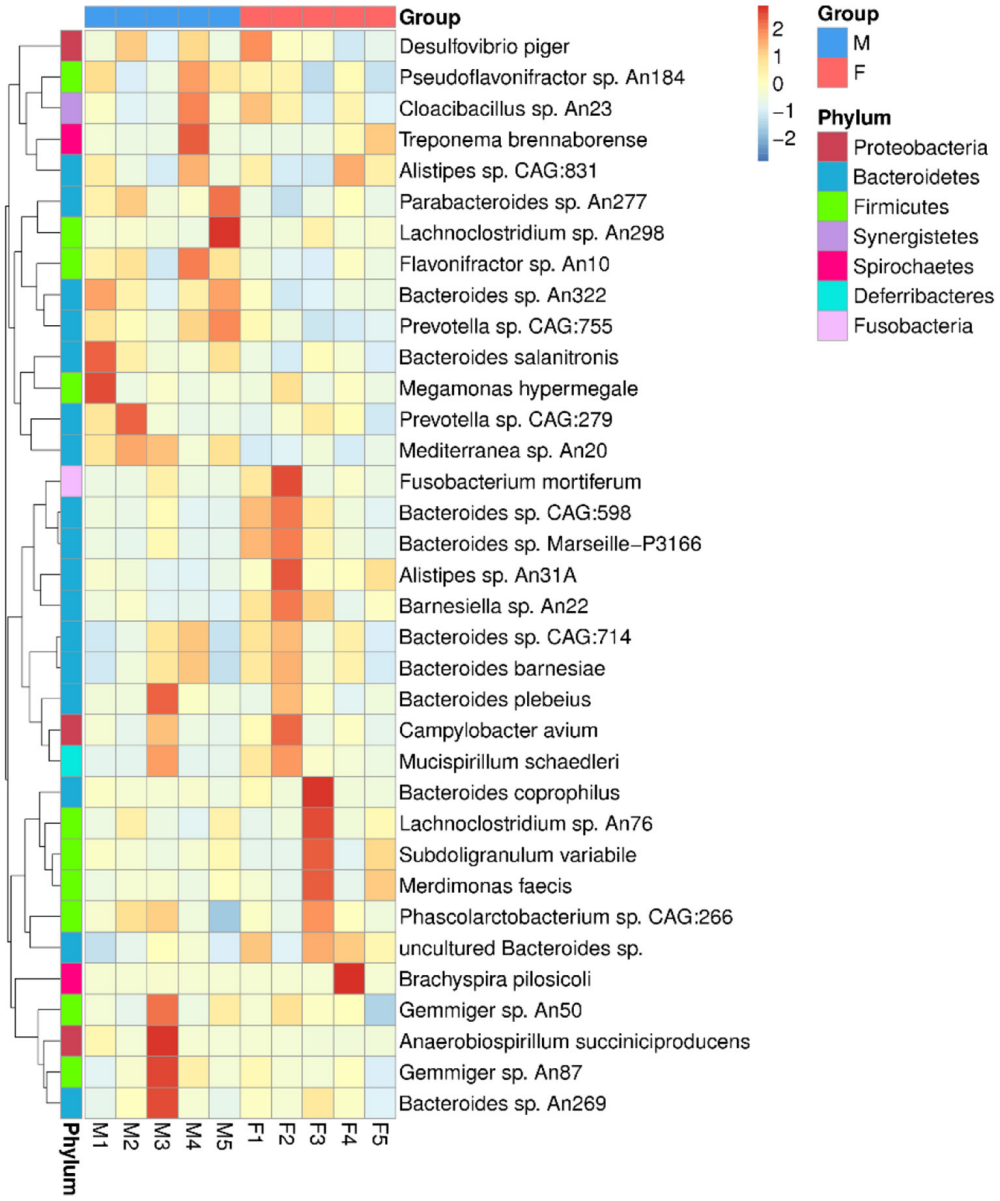
The top abundant 35 bacterial species clustered in the cecal samples of female (F) and male (M) Japanese quail (*Coturnix japonica*). The value corresponding to each block was the *Z* value of each row of species, represented the standardized relative abundance. The *Z* value was the difference between the relative abundance of samples in the classification and the average relative abundance of all samples, and then divided by the standard deviation of all samples.

The top species were *Alistipes* sp. CAG: 831, *Mediterranea* sp. An20, *Lachnoclostridium* sp. An298, *Fusobacterium mortiferum, Flavonifractor* sp. An10, *Phascolarctobacterium* sp. CAG:266, *Brachyspira pilosicoli, uncultured Bacteroides* sp., *Bacteroides barnesiae* and *Desulfovibrio piger* (Figure 1B). The abundances of the top 35 species per sample were used to generate a heat map. The species with higher concentrations were clustered in the samples (Figure 2).

**Figure 3:**
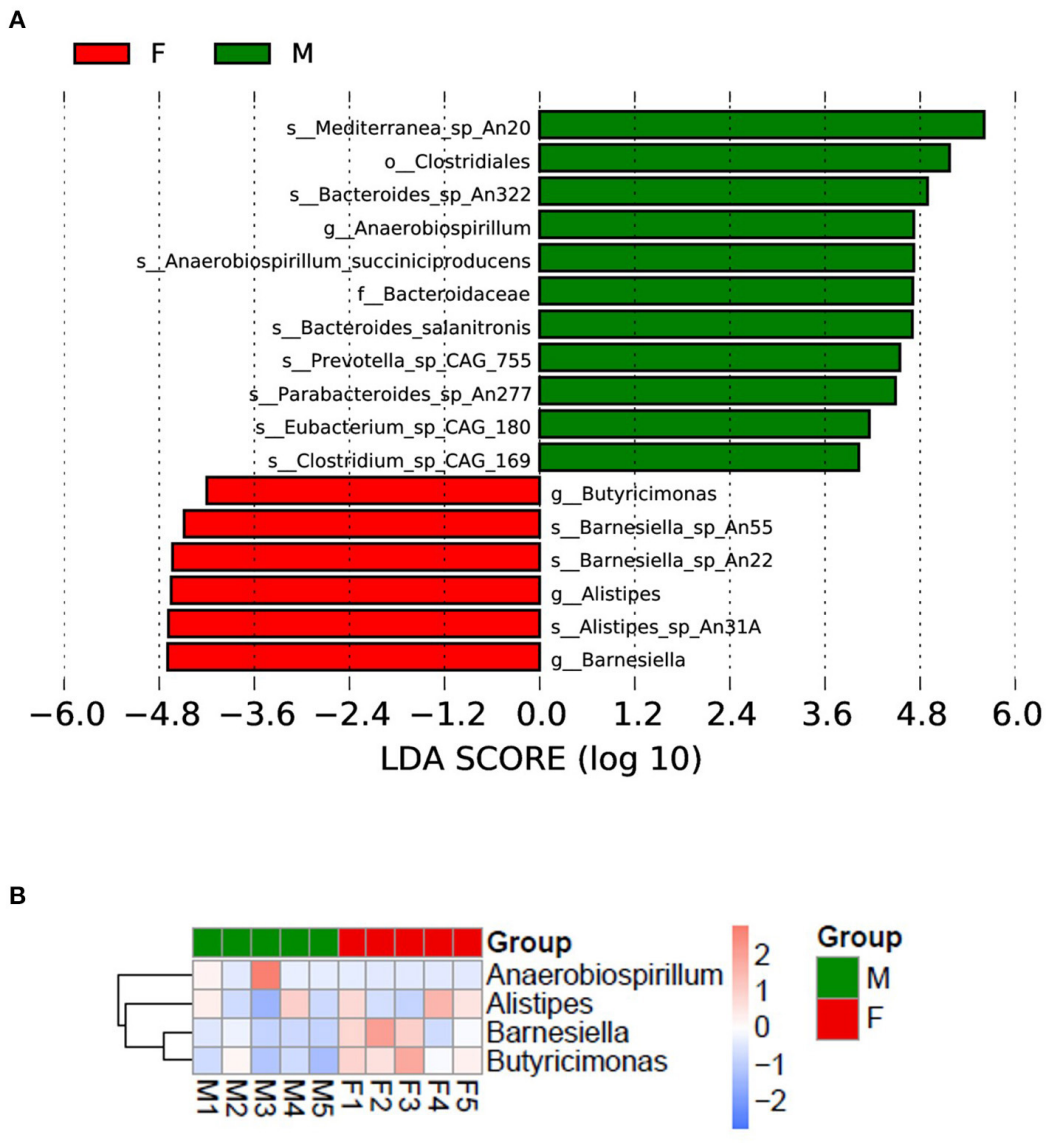
**(A)** Different bacterial species in female (F) and male (M) Japanese quail (*Coturnix japonica*); **(B)** different species clustered in four genera.

To investigate the different species in the male and female quail, we performed a LEfSe analysis, which yielded 17 differentially abundant species (Figure 3A). Eleven taxa were more abundant in the male quail: *Mediterranea* sp. An20, Clostridiales, *Bacteroides* sp. An322, *Anaerobiospirillum, Anaerobiospirillum succiniciproducens*, Bacteroidaceae, *Bacteroides salanitronis, Prevotella* sp. CAG:755, *_Parabacteroides* sp. An277, *Eubacterium* sp. CAG:180, and *Clostridium* sp. CAG:169. In the female quail, *Butyricimonas, Barnesiella* sp. An55, *Barnesiella* sp. An22, *Alistipes, Alistipes* sp. An31, and *Barnesiella* were more abundant. The data showed that the different species were clustered into four genera: *Anaerobiospirillum, Alistipes, Barnesiella* and *Butyricimonas* (Figure 3B).

### Gene Function Analysis

To obtain gene function information, the genes were analyzed separately via BLAST against the KEGG, EggNOG, CAZy, and CARD databases using DIAMOND software. For the functional annotation, 804,714 genes were annotated in the KEGG database, followed by 791,180 in EggNOG, 46,317 in CAZy, and 496 in CARD (Supplemental File 5). Of the 496 genes in CARD, 308 were for antibiotic resistance.

The KEGG database profile on the relative abundances of the gene functions suggested that the main activities of the genes identified were associated with metabolism, genetic information processing and environmental information at the first classification level. Genes related to the three terms of replication, recombination and repair, amino acid transport and metabolism, and cell wall/membrane/envelope biogenesis were the most abundant in EggNOG. Most genes matched in CAZy were associated with the functions of glycoside hydrolases and glycosyl transferases.

### KEGG

The metabolic pathway predictions provided a functional description of gut cell metabolism in Japanese quail: 164,216 genes were related to 132 unique pathways at the third classification level. This showed that the quail gut microbes had different activities related to functions such as the metabolism of carbohydrates (50,376 genes), amino acids (40,573 genes), vitamins (29,684 genes), nucleotides (27,397 genes), and energy (25,831 genes; Supplemental File 6).

### EggNOG

The BLAST results based on EggNOG showed that more unigenes were in the classes of amino acid transport and metabolism (55,478 genes) and carbohydrate transport and metabolism (54,316 genes), while a cluster of 43,338 genes was assigned to inorganic ion transport and metabolism.

### CAZy Database

CAZy classified 46,317 genes into six ontologies, including glycoside hydrolases (28,771 genes), glycosyl transferases (11,718 genes), carbohydrate-binding modules (4,055 genes), carbohydrate esterases (2,590 genes), polysaccharide lyases (857 genes), and auxiliary activities (7 genes).

### ANOSIM and LEfSe Analysis

**Figure 4:**
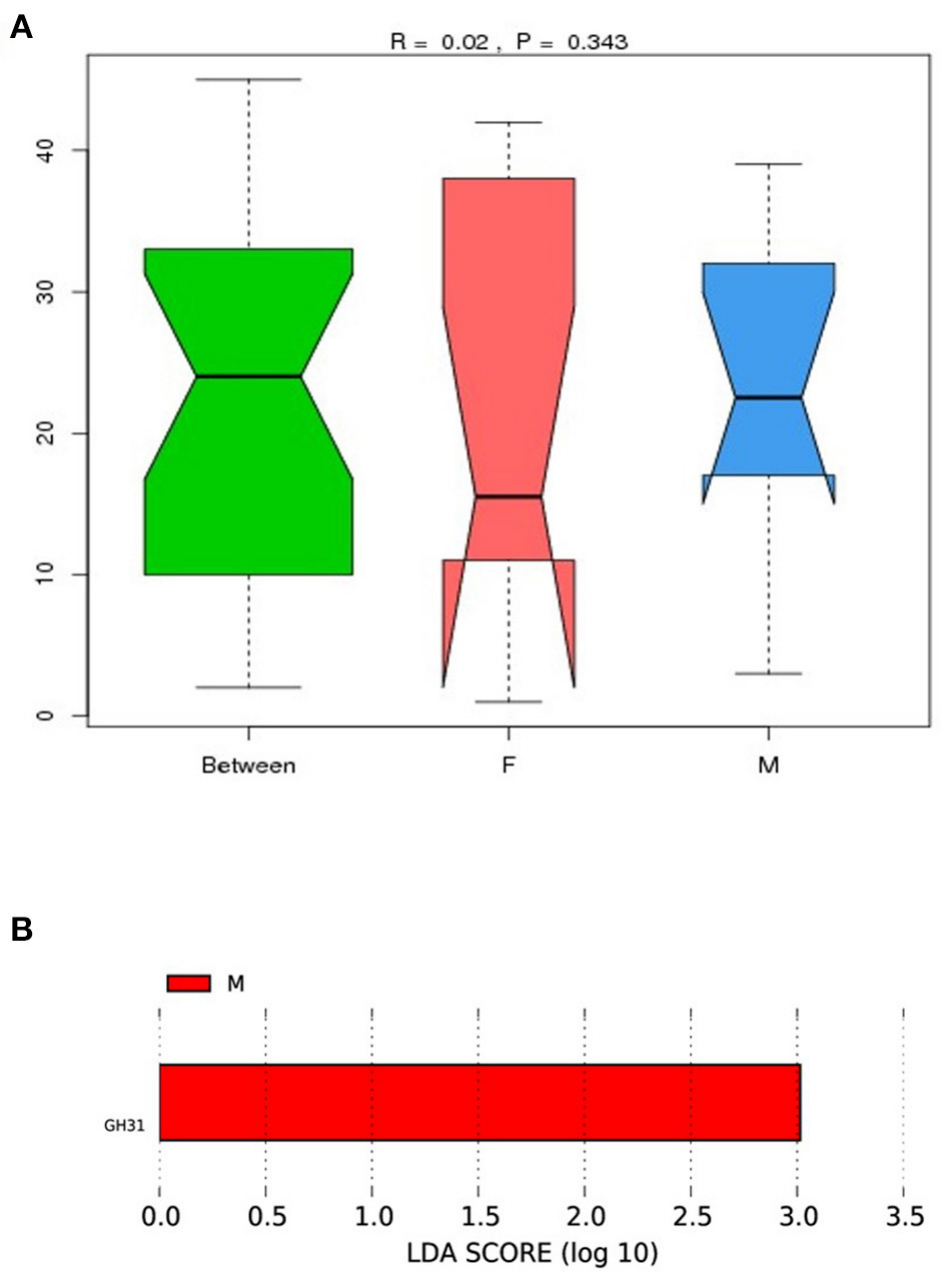
**(A)** Analysis of similarity (ANOSIM) between female (F) and male (M) Japanese quail (*Coturnix japonica*) based on the CAZy database. The horizontal axis represents the grouping information; the vertical axis indicates the distance data, and “Between” is the combined information for both groups. The median line for “Between” being higher than those of the other two groups suggests that the groupings were appropriate. The *R*-value was between −1 and 1 and >0, indicating a significant difference between the groups. The *P*-value represents the reliability of the statistical analysis, and *P* < 0.05 indicates statistical significance; **(B)** Different functional capacities of gut microbiome based on Carbohydrate-Active Enzymes (Cazy) analysis between female (F) and male (M) Japanese quail (Coturnix japonica).

To examine the similarities between the male and female quail, we analyzed the differences in relative gene abundances and their corresponding functions in the ten quail. CAZy results showed differences between the groups (Figure 4A); however, the differences between the measurement data were not statistically significant. The results suggested that the abundances of gut microbes that produce carbohydrate-active enzymes may differ between female and male quail.

To investigate the difference in functional capacities of gut microbiome between female and male quail, we performed a LEfSe analysis, the significant difference was yielded based on the result of Cazy analysis. The genes of GH31 annotation was significantly different between female and male quail (Figure 4B).

### Analysis of Antibiotic-Resistance Genes and Bacteria

**Figure 5:**
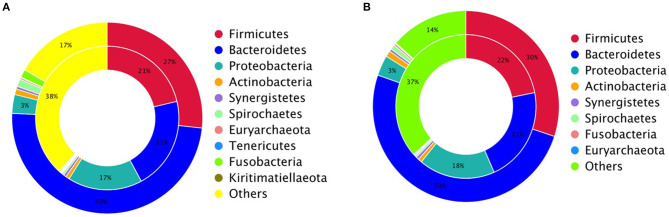
Analysis of unique antibiotic-resistant bacteria in Japanese quail (*Coturnix japonica*): **(A)** predominant bacterial hosts of antibiotic-resistance genes (ARGs) in the female quail; **(B)** predominant bacterial ARG hosts in the male quail.

In contrast to the above results, fewer genes were related in the CARD database, which matched 496 genes; 308 of these were antibiotic-resistance genes (ARGs), with an abundance of 2.98 × 10^−4^. Of these genes, 110 were found in the ten quail (Supplemental File 7). The most abundant genes included *tetQ, tetW, ermF, adeF, tetW/N/W, lnuC, sul2, tet40, tetO*, and *APH3-IIIa*. Species information was collected for each ARG from each quail. These ARGs were harbored by diverse phyla, including Bacteroidetes, Firmicutes, and Euryarchaeota (Figure 5).

Analysis of the ARG distributions showed that the female quail had 147 common genes and 24 unique genes, and the male quail had 124 common genes and ten unique genes (Supplemental Figure 4 and Supplemental File 7). Bacteroidetes were the predominant ARG hosts in the female quail; Euryarchaeota were the dominant ARG hosts in the male quail. Tenericutes and Kiritimatiellaeota were detected only in the female quail (Figure 5).

## Discussion

We conducted an Illumina short-read-based gene-annotation sequencing analysis of metagenomic DNA taken from the ceca of ten Japanese quail, including five female quail and five male quail, to obtain a catalog of bacterial gene communities in adult quail. To our knowledge, this is the first analysis of the gut microbiota gene compositions for this species. The results from the gene annotation against the MicroNR database revealed several abundant groups at the phylum level, including Bacteroidetes, Firmicutes, Proteobacteria, Spirochaetes and Fusobacteria. 16S rRNA gene sequencing revealed similar results for the ileal microbiotas of this species (15). Additionally, Tenericutes was a major phylum in cecal samples from Japanese quail (12); however, the abundance of Firmicutes was higher than that of Bacteroidetes (12, 15), both of which likely dominated the cecal microbiota (11, 12, 15). Other studies have shown that higher abundances of Firmicutes could lead to more efficient absorption of calories from the digesta (48, 49), which may be related to a higher abundance of Firmicutes in the ileum and a lower abundance in the cecum. *Bacteroides* spp. play key roles in immunoregulatory abilities and help provide valuable nutrients and energy to the host by breaking down food (50, 51). Bacteroidetes may also contribute to disease resistance in the host. Moreover, *Bacteroides* can degrade indigestible fiber in chicken guts (20), and the enriched *Bacteroides* from the quail ceca was consistent with substantial microbial fermentation in the hindgut.

Our gene function prediction results showed that the microbial species in the quail ceca contributed to metabolic functions involving production of energy and nutrients by digesting food, showing that the cecal microbiota is an important energy supplier for the host. The GH31 might contribute to metabolic functions, since the functional capacity analysis of gut microbiome found the GH31 annotation was significantly different between female and male group. These results reflected the important role of the cecal microbiota in some feed-related traits, such as phosphorus utilization, daily gain, feed intake and feed per gain ratio reported in Japanese quail as assessed by mixed linear models (15). Our data revealed genes belonging to subspecies of *Bacillus* and *Enterococcus*, which are considered probiotics in chickens and Japanese quail (17, 18). *Bacillus* exerts positive effects on several feed-related traits.

Our analysis identified ~3.4 million comprehensive Japanese quail gut microbial genes. Animal gut microbiotas, including those of humans (31, 32, 52), dogs (53), monkeys (54), mice (55), rats (56, 57), chickens (58), and pigs (25, 59), are receiving increasing attention worldwide for their important contributions to host nutrition and health. The size of the Japanese quail gene catalog was smaller than those of chickens (9.04 million genes), humans (9.9 million genes) and pigs (7.7 million genes) (58), possibly owing to the limited samples from only the lumens of the ceca, which do not contain gene information on the microbiome in the foregut. From our data, means of 88.74, 0.59, 0.0364, and 0.203% of the genes were annotated to bacteria, viruses, eukaryotes and archaea, respectively. No viral genes have been found in chicken gut microbiotas (58).

Metagenomic sequencing identified 308 ARGs, of which, the dominant genes were *tetQ, tetW, ermF, adeF*, t*etW/N/W, lnuC, sul2, tet40, tetO*, and *APH3-IIIa*. Some ARGs, such as *tetM, vanX* and *bla*, existed before the advent of antibiotic use (60). The ARG abundances in the quail (*P* = 2.98 × 10^−4^) in our study were lower than those reported in the pigs (61) and were mainly distributed in Bacteroidetes, Firmicutes and Euryarchaeota. High-throughput next-generation sequencing enables detecting the presence of broad-spectrum antibiotic resistance in animal gut fecal resistomes. To date, numerous studies on ARGs in regard to existing and emerging antibiotic-resistance threats have been reported for humans, sheep, chickens, pigs, and cows (62–64). Because of the intensive link between the spread of ARGs and human health, increasing knowledge of ARGs is important for addressing potential threats to human health.

Our results showed that female quail had relatively high microbial species abundances, whereas male quail had a higher microbiome diversity (Chao1 index) (12). The differences between the male and female quail were analyzed, and 17 species that were clustered into four genera (*Anaerobiospirillum, Alistipes, Barnesiella*, and *Butyricimonas*) from four families (Succinivibrionaceae, Rikenellaceae, Barnesiellaceae, and Odoribacteraceae) were differentially abundant. Compared with the families Lactobacillaceae and Catabacteriaceae, which were differentially abundant in the large intestines of male and female Japanese quail (12), our results revealed significant differences within the quail. The differences in these microbiomes may be related to the differences in growth potential between male and female quail. Bacterial communities also differ significantly between male and female broiler chicken ceca. Male chicken ceca were enriched with *Bacteroides*, whereas female chicken ceca were enriched with *Clostridium* and *Shigella* (20). These detected bacterial species varied between chickens (65) and quail. However, some of our results are preliminary, and the functions of these gut microbes require further study.

In summary, our study provides the first reference gene catalog for the Japanese quail gut microbiome, which will be an important addition to animal gut metagenomics. Metagenomic analysis will contribute to future studies on the differences in the mechanisms of feed-related quantitative traits and the gut microbiome in quail. Our results also help explain why female quail exhibit greater muscle growth potential than do male quail.

## Data Availability Statement

The original contributions presented in the study are publicly available. This data can be found here: SRA accession: SUB9360165.

## Ethics Statement

The animal study was reviewed and approved by Animal Care Committee of Nanchang Normal University (Nanchang, China).

## Author Contributions

J-EM, X-WX, and J-GX carried out the design and wrote the manuscript. J-SG, Y-FL, QX, and Y-BY helped to revise the draft manuscript. JL, X-NZ, Y-WT, and W-TS help to collect the samples. X-TT, Z-FW, and MZ help to analyze the data. C-YZ sorted some of the tables. X-QZ provided some effective suggestions. Y-SR designed the study and revised the manuscript. All authors read and approved the final manuscript.

## Funding

This work was supported by the Research Fund of Nanchang Normal University for Doctors (No. NSBSJJ2019010), the Natural Science Foundation of Education Department in Jiangxi Province (No. GJJ191131), the ShuangQian planning Projects of Jiangxi Province, the Open Project of Guangdong Provincial Key Lab of Agro-Animal Genomics and Molecular Breeding, and the China Agriculture Research System (CARS-41- G03).

## Conflict of Interest

The authors declare that the research was conducted in the absence of any commercial or financial relationships that could be construed as a potential conflict of interest.

## Publisher's Note

All claims expressed in this article are solely those of the authors and do not necessarily represent those of their affiliated organizations, or those of the publisher, the editors and the reviewers. Any product that may be evaluated in this article, or claim that may be made by its manufacturer, is not guaranteed or endorsed by the publisher.
