# Supplementary material for: Metagenomic Analysis Identifies Sex-Related Cecal Microbial Gene Functions and Bacterial Taxa in the Quail
Source: Front Vet Sci. 2021 Oct 1;8:693755. doi: 10.3389/fvets.2021.693755 (PMC8517240; doi:10.3389/fvets.2021.693755)
Supplement: Supplementary file 8 [file Table_8.DOCX]

Table S1 The detail information of the samples

|  | Age | Weight | Gender | Physiological state |
| --- | --- | --- | --- | --- |
| F-1 | 72 days | 131.7g | Female | healthy |
| F-2 | 72 days | 132.4g | Female | healthy |
| F-3 | 72 days | 138.9g | Female | healthy |
| F-4 | 72 days | 136.8g | Female | healthy |
| F-5 | 72 days | 123.8g | Female | healthy |
| M-1 | 72 days | 107.5g | Male | healthy |
| M-2 | 72 days | 102.1g | Male | healthy |
| M-3 | 72 days | 111.1g | Male | healthy |
| M-4 | 72 days | 102.7g | Male | healthy |
| M-5 | 72 days | 104.3g | Male | healthy |
